# First-line biologic therapy with tumor necrosis factor inhibitors for psoriatic arthritis: a prospective observational study

**DOI:** 10.1590/1516-3180.2021.0434.R1.22022022

**Published:** 2022-08-29

**Authors:** Michael Ruberson Ribeiro da Silva, Jéssica Barreto Ribeiro dos Santos, Adriana Maria Kakehasi, Alessandra Maciel Almeida, Pedro Ricardo Kömel Pimenta, Juliana Alvares-Teodoro, Francisco de Assis Acurcio

**Affiliations:** I PhD. Pharmacist and Adjunct Professor, Department of Pharmacy and Nutrition, Universidade Federal do Espírito Santo (UFES), Alegre (ES), Brazil.; II PhD. Pharmacist and Adjunct Professor, Department of Pharmacy and Nutrition, Universidade Federal do Espírito Santo (UFES), Alegre (ES), Brazil.; III PhD. Physician and Associate Professor, Department of Locomotor System, Universidade Federal de Minas Gerais (UFMG), Belo Horizonte (MG), Brazil.; IV PhD. Dentist and Adjunct Professor, Instituto de Pesquisa e Pós-Graduação em Ciências Médicas, Faculdade de Ciências Médicas de Minas Gerais (FCMMG), Belo Horizonte (MG), Brazil.; V MD. Pharmacist and Researcher Department of Social Pharmacy, Universidade Federal de Minas Gerais (UFMG), Belo Horizonte (MG), Brazil.; VI PhD. Pharmacist and Adjunct Professor, Department of Social Pharmacy, Universidade Federal de Minas Gerais (UFMG), Belo Horizonte (MG), Brazil.; VII PhD. Physician And Full Professor, Department of Social Pharmacy, Universidade Federal de Minas Gerais (UFMG), Belo Horizonte (MG), Brazil.

**Keywords:** Arthritis, psoriatic, Comparative effectiveness research, Adalimumab, Etanercept, Observational study [publication type], Minimal clinically important difference, Spondylarthritis, TNF inhibitors, Good clinical response, Quality of life, Safety

## Abstract

**BACKGROUND:**

Psoriatic arthritis (PsA) is a chronic inflammatory disease that affects multiple joints. It is associated with psoriasis and treated with synthetic and biologic drugs.

**OBJECTIVE:**

The objective of this study was to assess the outcomes of patients who received biologic therapy with tumor necrosis factor (TNF) inhibitors in terms of effectiveness, safety, functionality, and quality of life.

**DESIGN AND SETTING:**

A prospective observational study was performed at a single center in Belo Horizonte, Brazil.

**METHODS:**

Patients with PsA who received their first TNF inhibitor treatment were followed up for 12 months. Disease activity was measured using the Bath Ankylosing Spondylitis Disease Activity Index (BASDAI) and Clinical Disease Activity Index (CDAI). Functionality was measured using the Health Questionnaire Assessment (HAQ), and quality of life was evaluated using the European Quality of Life Five Dimensions (EQ-5D). Multiple linear regression was used to identify predictors of the clinical response at 12 months.

**RESULTS:**

A total of 143 patients treated with adalimumab or etanercept were evaluated. Most of the clinical measures were significantly improved at 12 months. However, 31%–51% of the patients did not achieve good clinical control. No differences were observed between adalimumab and etanercept, except for poor functionality at 12 months among patients treated with etanercept. The main predictors of a worse clinical response were female sex, etanercept use, poor functionality, or lower quality of life at baseline. The main adverse reactions were alopecia, headache, injection site reaction, sinusitis, flu, dyslipidemia, and infections.

**CONCLUSION:**

TNF inhibitor therapy was effective and safe. However, despite improvements in clinical measures, most patients did not achieve satisfactory control of the disease.

## INTRODUCTION

Psoriatic arthritis (PsA) is a chronic inflammatory disease that is usually seronegative for rheumatoid factor and has diverse clinical manifestations.^1^ The different clinical features are challenging for physicians in terms of diagnosis and treatment.^1,2^ Delayed diagnosis of PsA is associated with irreversible damage, and streamlined early treatment with disease-modifying antirheumatic drugs (DMARDs) can slow disease progression and improve physical function and quality of life.^1,3^

In Brazil, the treatment of PsA is covered by the Unified Health System (Sistema Único de Saúde, SUS), a national public health system subsidized by taxes, which provides primary, out-patient, and hospital care in addition to drugs and other health technologies for comprehensive treatment.^4,5^ Around 210 million people are covered, of which 75% are exclusively assisted by the SUS. PsA patients are attended to by doctors from the public and private sectors (SUS and non-SUS). Their medication is covered by the SUS, health plans, or out-of-pocket expenses. However, the supply of biologic DMARDs (bDMARDs) is almost entirely realized by SUS pharmacies because of the high cost of these drugs to PsA patients.^4,5^

The drugs available through the SUS include nonsteroidal anti-inflammatory drugs (NSAIDs), glucocorticoids, and conventional synthetic, biologic, and target-specific synthetic DMARDs.^6^

NSAIDs and glucocorticoids are usually used to control disease symptoms, such as pain and swelling. DMARDs are immunosuppressive and immunomodulatory agents that can modify the natural course of the disease, including delays in clinical or radiographic progression. bDMARDs, including tumor necrosis factor-alpha inhibitors (adalimumab, etanercept, infliximab, golimumab, and certolizumab) and interleukin-17 inhibitors (secukinumab), are usually prescribed after the failure of conventional synthetic disease-modifying antirheumatic drugs (csDMARDs).^6^

The advent of tumor necrosis factor inhibitors (TNFis) has resulted in a substantial improvement in the treatment of PsA refractory to csDMARDs, and the efficacy of these agents has been demonstrated in randomized controlled trials.^7^ However, limited head-to-head studies have compared the clinical efficacy of these drugs.^8^

Despite the benefits observed with biologic TNFis in the last few years, approximately 40% of patients discontinued treatment in the 12 months of follow-up. In addition, the substantial economic impact of TNFi therapy on health systems was observed, accounting for 90% of the PsA treatment cost.^4^ Therefore, a real-world evaluation is warranted.

Observational studies are instrumental in complementing the scientific evidence of efficacy and safety provided by randomized controlled trials.^9^ Furthermore, in the absence of head-to-head randomized controlled trials comparing two or more bDMARDs, observational studies with a common drug comparator can be used to evaluate and compare these drugs in clinical practice.^10^

## OBJECTIVE

The objective of this study was to evaluate the outcomes of patients diagnosed with PsA in Brazil who received TNFi therapy in terms of effectiveness, functionality, quality of life, and safety.

## METHODS

### Type of study, patient characteristics, and data collection

An open, prospective, observational study of patients with PsA treated through the SUS was performed at a single center in Belo Horizonte from January 2012 to July 2019. This center is responsible for supplying drugs to approximately 320 patients with PsA.

The eligibility criteria were 18 years of age or older, diagnosis of PsA according to the Classification Criteria for Psoriatic Arthritis (CASPAR), and use of TNFis.^11^ Patients treated with golimumab and infliximab were excluded due to the small number of patients. Furthermore, patients who were unable to visit the pharmacy regularly to receive their medications were excluded from the study.

Follow-up started on the first dispensation of TNFis, and the patients were reassessed at approximately 6 and 12 months.

A standardized research form was used, which was developed and tested previously. Sociodemographic characteristics, such as age, sex, education, marital status, and self-declared ethnicity, were recorded. Data on disease duration, current and previous PsA drug use, comorbidities, adverse reactions, disease activity, functionality, and quality of life were also collected. Interviews were conducted face-to-face with the patients by a team of researchers comprising pharmacists and graduate and undergraduate pharmacy students. The researchers were trained in a specialized rheumatology center where it was possible to follow up on the care of patients with PsA.

The Research Ethics Committee of the Universidade Federal de Minas Gerais (UFMG) approved this study (opinion number 0069.0.203.000-11) on May 26, 2011. All of the patients signed a consent form.

### Outcomes

Disease activity was measured using the Bath Ankylosing Spondylitis Disease Activity Index (BASDAI) and the Clinical Disease Activity Index (CDAI).^12–15^ The BASDAI assesses axial involvement, and the CDAI evaluates peripheral involvement. Functionality was measured using the Health Questionnaire Assessment (HAQ), and quality of life was evaluated using the European Quality of Life Five Dimensions Questionnaire (EQ-5D); both have versions that have been validated for Brazil.^12,13^

A good clinical response (GCR) was defined as a BASDAI < 4 and a CDAI ≤ 10. Additionally, the outcome for a BASDAI reduction ≥ 2 points or 50% was assessed.^13,14^ A minimal clinically important difference (MCID) was defined as an improvement of ≥ 0.05 for quality of life according to the EQ-5D and a reduction of ≥ 0.35 for HAQ functionality.^16,17^ The GCR and MCID were defined as the proportion of clinical response. Subgroup analysis was performed to verify the effect of the main comorbidities on disease activity, functionality, and quality of life. The occurrence of drug adverse reactions was self-reported.

### Statistical analysis

The sample size was estimated considering the MCID for the HAQ and EQ-5D outcomes for paired samples (baseline and end of follow-up). A difference of 0.35 (Δ = 0.35), a standard deviation of 0.70, a correlation between paired samples of 0.60, a statistical significance of 5% (α = 0.05), and a power test of 80% (β = 0.80) were used for the HAQ outcome, which indicated a minimal sample of 28 patients per group, for a total of 56 patients. A difference of 0.05 (Δ = 0.05), a standard deviation of 0.15, a correlation between paired samples of 0.60, a statistical significance of 5% (α = 0.05), and a power test of 80% (β = 0.80) were used for the EQ-5D outcome, which indicated a minimal sample of 59 patients per group, for a total of 118 patients. Therefore, a sample of 118 patients was considered for this study.

Descriptive analysis was performed using the frequency distribution, mean, and standard deviation. An independent t-test for two independent groups and a paired t-test for two paired groups were used for continuous variables. Pearson's chi-squared test was used for categorical variables.

Multiple imputations addressed missing data. A predictive mean matching method was adopted considering the monotonic pattern observed in the missing data; missing data at 6 months were also missing at 12 months.^18.19^

Nearest neighbor matching was used to evaluate the comparative effectiveness, functionality, and quality of life between TNFis.^20^ Therefore, patients were paired according to similar characteristics at baseline. A significance level of 5% was used for comparative analysis.

Multiple linear regression with a 95% confidence interval (CI) was used to identify predictive factors for clinical response according to the CDAI, BASDAI, HAQ, and EQ-5D at 12 months of follow-up. Sex, age, education, marital status, ethnicity, disease duration, comorbidity, disease activity, functionality, quality of life, bDMARD use, NSAID use, csDMARD use, and glucocorticoid use were considered independent variables. A significance level of 5% (P < 0.05) was used for these analyses.

Statistical analyses were performed using Stata version 16.1 (StataCorp, College Station, Texas, United States).

## RESULTS

### Baseline characteristics

A total of 143 PsA patients were included. Loss to follow-up (withdrawal from the study) was observed for 21 patients (14.7%) at 6 months and 92 patients (35.7%) at 12 months. Lack of effectiveness (23.1%) and adverse reactions (14.1%) were the main causes of the loss to follow-up.

The mean age was 51.13 years (standard deviation = 12.23), and the mean duration of the disease was 5.09 years (6.90). Most patients were white (53.8%), married (61.0%), and educated up to the high school level (69.5%) (Table 1). Of the 143 patients, 91 patients (63.6%) were treated with adalimumab, and 52 patients (36.3%) were treated with etanercept. In addition, 58 (40.6%), 34 (23.8%), and 36 (25.2%) patients concomitantly used csDMARDs, NSAIDs, and glucocorticoids, respectively. At baseline, the mean CDAI, BASDAI, HAQ, and EQ-5D scores were 22.79 (16.29), 5.38 (2.42), 1.22 (0.73), and 0.65 (0.18), respectively (Table 1).

**Table 1:** Baseline sociodemographic and clinical characteristics of psoriatic arthritis patients

Variable		Adalimumab (91)	Etanercept (52)	Total (143)	P value
**Sex**, n (%)					0.386
	Female	51 (56.0)	33 (63.5)	84 (58.7)	
	Male	40 (44.0)	19 (36.5)	59 (41.3)	
**Age**, mean (SD)		50.92 (11.89)	51.50 (12.90)	51.13 (12.23)	0.787
**Duration of disease**, mean (SD)		5.36 (7.27)	4.61 (6.25)	5.09 (6.90)	0.532
**Ethnicity, n** (%)					0.92
	White	48 (52.8)	29 (55.8)	77 (53.8)	
	Brown	31 (34.1)	16 (30.8)	47 (32.9)	
	Black	12 (13.2)	7 (13.5)	19 (13.3)	
**Marital status**, n (%)					0.162
	Single	17 (19,1)	17 (32.7)	34 (24.1)	
	Married	59 (66.3)	27 (51.9)	86 (61.0)	
	Other	13 (14.6)	8 (15.4)	21 (14.9)	
**Education**, n (%)					0.066
	≤ Elementary	27 (30.3)	12 (23.1)	39 (27.7)	
	> Elementary to ≤ high school	41 (46.1)	18 (34.6)	59 (41.8)	
	Undergraduate	21 (23.6)	22 (42.3)	43 (30.5)	
**Comorbidity**, n (%)		68 (74.7)	40 (76.9)	108 (75.5)	0.769
**Concomitant csDMARDs**, n (%)		43 (47.2)	15 (28.9)	58 (40.6)	**0.031**
**Concomitant NSAIDs**, n (%)		25 (27.5)	9 (17.3)	34 (23.8)	0.170
**Concomitant glucocorticoids**, n (%)		27 (29.7)	9 (17.3)	36 (25.2)	0.101
**CDAI**, mean (SD)		23.74 (16.64)	21.13 (15.69)	22.79 (16.29)	0.357
**BASDAI**, mean (SD)		5.21 (2.46)	5.68 (2.39)	5.38 (2.42)	0.266
**HAQ**, mean (SD)		1.23 (0.74)	1.21 (0.71)	1.22 (0.73)	0.873
**EQ-5D**, mean (SD)		0.64 (0.18)	0.66 (0.18)	0.65 (0.18)	0.507

n = number of patients; SD = standard deviation; csDMARDs = conventional synthetic disease-modifying antirheumatic drugs; NSAIDs = nonsteroidal anti-inflammatory drugs; CDAI = Clinical Disease Activity Index; BASDAI = Bath Ankylosing Spondylitis Disease Activity Index; HAQ = Health Assessment Questionnaire; EQ-5D = European Quality of Life Five Dimensions.

The main comorbidities reported were hypertension (n = 43; 30.1%), dyslipidemia (n = 34; 23.8%), depression (n = 28; 19.6%), and diabetes mellitus (n = 22; 15.4%). Other reported comorbidities were gastritis (n = 11; 7.7%), hypothyroidism (n = 10; 7.0%), anxiety (n = 9; 6.3%), and fibromyalgia (n = 6; 4.2%).

### Effectiveness, functionality, and quality of life

**Figure 1:**
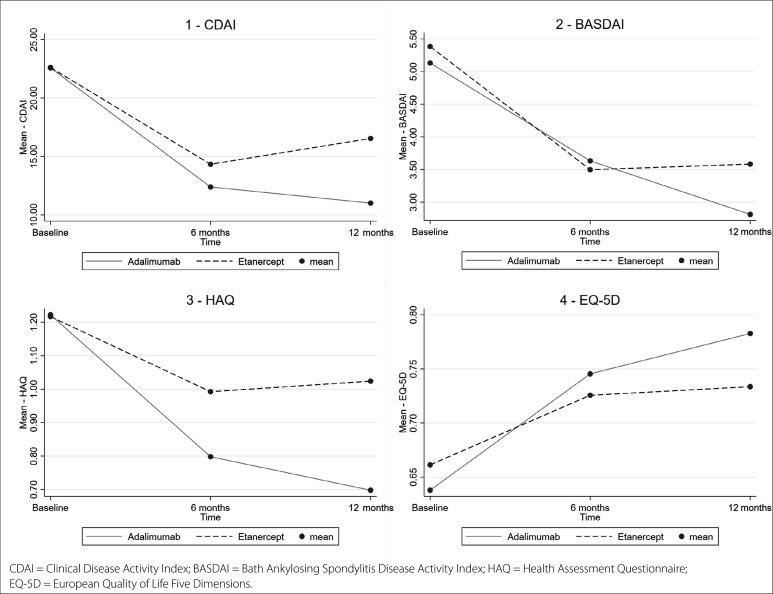
Disease activity, functionality, and quality of life at baseline, 6 months, and 12 months for patients treated with adalimumab or etanercept.

All clinical measures of disease activity, functionality, and quality of life were significantly improved at 6 and 12 months compared with the baseline among patients treated with adalimumab (P < 0.001). Most clinical measures also showed a statistically significant reduction at 6 and 12 months compared with the baseline among patients treated with etanercept, except for a borderline value in the HAQ at 12 months (Table 2 and Figure 1).

**Table 2:** Effectiveness, functionality, and quality of life at baseline, 6 months, and 12 months for patients who received TNFi therapy

Variable		CDAI									
TNFi		Baseline			6 months				12 months		
		mean	SD	mean	SD	Δ	P value*	mean	SD	Δ	P value**
**Overall**		22.79	16.29	13.29	12.94	−9.50	< 0.001	13.45	12.85	−9.35	< 0.001
	Adalimumab	23.74	16.64	13.42	12.77	−10.32	< 0.001	11.73	11.00	−12.01	< 0.001
	Etanercept	21.13	15.69	13.07	13.35	−8.06	< 0.001	16.44	15.24	−4.69	0.033
**Variable**		**BASDAI**									
**TNFi**		**Baseline**			**6 months**				**12 months**		
		**mean**	**SD**	**mean**	**SD**	**Δ**	**P value** *	**mean**	**SD**	**Δ**	**P value** **
**Overall**		5.38	2.42	3.59	2.42	−1.79	< 0.001	3.06	2.08	−2.32	< 0.001
	Adalimumab	5.21	2.44	3.67	2.40	−1.54	< 0.001	2.82	2.07	−2.39	< 0.001
	Etanercept	5.68	2.39	3.45	2.47	−2.23	< 0.001	3.47	2.03	−2.21	< 0.001
**Variable**		**Functionality (HAQ)**									
**TNFi**		**Baseline**			**6 months**				**12 months**		
		**mean**	**SD**	**mean**	**SD**	**Δ**	**P value** *	**mean**	**SD**	**Δ**	**P value** **
**Overall**		1.22	0.73	0.87	0.68	−0.35	< 0.001	0.82	0.62	−0.40	< 0.001
	Adalimumab	1.23	0.74	0.79	0.63	−0.44	< 0.001	0.69	0.55	−0.54	< 0.001
	Etanercept	1.21	0.71	1.01	0.73	−0.20	0.020	1.05	0.67	−0.16	0.055
**Variable**		**Quality of life (EQ-5D)**									
**TNFi**		**Baseline**			**6 months**				**12 months**		
		**mean**	**SD**	**mean**	**SD**	**Δ**	**P value** *	**mean**	**SD**	**Δ**	**P value** **
**Overall**		0.65	0.15	0.73	0.18	0.09	< 0.001	0.76	0.15	0.11	< 0.001
	Adalimumab	0.64	0.18	0.74	0.18	0.10	< 0.001	0.77	0.15	0.13	< 0.001
	Etanercept	0.66	0.18	0.73	0.18	0.07	0.003	0.73	0.17	0.07	0.004

TNFi = tumor necrosis factor inhibitor; CDAI = Clinical Disease Activity Index; SD = standard deviation; BASDAI = Bath Ankylosing Spondylitis Disease Activity Index; HAQ = Health Assessment Questionnaire; EQ-5D = European Quality of Life Five Dimensions.

* P value based on 6 months versus baseline;

** P-value based on 12 months versus baseline.

Following nearest neighbor matching, no differences were observed between TNFis in a comparative effectiveness analysis, except for poor functionality according to the HAQ at 12 months among patients treated with etanercept compared with those treated with adalimumab (Table 3).

**Table 3:** Comparative effectiveness of TNFi therapy analyzed by nearest-neighbor matching

Outcome	Period	ATE (SE)	P value
CDAI	6 months	−1.69 (2.61)	0.518
CDAI	12 months	4.37 (2.61)	0.094
BASDAI	6 months	−0.53 (0.47)	0.256
BASDAI	12 months	0.72 (0.42)	0.083
HAQ	6 months	0.18 (0.14)	0.199
HAQ	12 months	0.31 (0.12)*	0.008*
EQ-5D	6 months	−0.01 (0.03)	0.855
EQ-5D	12 months	−0.04 (0.03)	0.191

TNFi = tumor necrosis factor inhibitor; ATE = average treatment effect; SE = standard error; CDAI = Clinical Disease Activity Index; BASDAI = Bath Ankylosing Spondylitis Disease Activity Index; HAQ = Health Assessment Questionnaire; EQ-5D = European Quality of Life Five Dimensions.

Balance variables: conventional synthetic disease-modifying antirheumatic drugs (csDMARDs), nonsteroidal anti-inflammatory drugs (NSAIDs), glucocorticoids, education, and marital status (P < 0.20 at baseline).

* P < 0.05.

In terms of the proportion of GCR and MCID, minimal differences were observed between TNFis. The overall GCR was 49.0% according to the CDAI and 69.2% according to the BASDAI at 12 months. The overall MCID was 59.4% according to the HAQ and 63.6% according to the EQ-5D at 12 months. The results for each TNFi are presented in Table 4.

**Table 4:** Proportion of patients who achieved a good clinical response and minimal clinically important difference at 6 and 12 months for each TNFi

Variable		Adalimumab (91)	Etanercept (52)	TNFi total (143)	P value
Outcome	Period	n (%)	n (%)	n (%)	
CDAI	6 months	45 (49.4)	26 (50.0)	71 (49.6)	0.950
CDAI	12 months	47 (51.6)	23 (44.2)	70 (49.0)	0.393
BASDAI*	6 months	51 (56.0)	33 (63.5)	84 (58.8)	0.386
BASDAI*	12 months	67 (73.6)	32 (61.5)	99 (69.2)	0.132
BASDAI**	6 months	43 (47.2)	30 (57.7)	73 (51.0)	0.230
BASDAI**	12 months	53 (58.2)	30 (57.7)	83 (58.0)	0.949
HAQ	6 months	55 (60.4)	23 (44.2)	78 (54.6)	0.061
HAQ	12 months	59 (63.7)	27 (51.9)	85 (59.4)	0.166
EQ-5D	6 months	54 (59.3)	28 (53.8)	82 (57.3)	0.523
EQ-5D	12 months	62 (68.1)	29 (55.8)	91 (63.6)	0.139

TNFi = tumor necrosis factor inhibitor; CDAI = Clinical Disease Activity Index; BASDAI = Bath Ankylosing Spondylitis Disease Activity Index; HAQ = Health Assessment Questionnaire; EQ-5D = European Quality of Life Five Dimensions.

* BASDAI < 4 points;

** BASDAI reduction of 50% or ≥ 2 points.

### Comorbidities

The main comorbidities were hypertension (n = 62; 30.2%), dyslipidemia (n = 47; 22.9%), depression (n = 37; 18.0%), diabetes (n = 29; 14.1%), gastritis (n = 15; 7.3%), hypothyroidism (n = 13; 6.3%), fibromyalgia (n = 9; 4.4%), anxiety (n = 9; 4.4%), arthrosis (n = 7; 3.4%), obesity (n = 7; 3.4%), and herniated disc (n = 6; 2.9%). According to the CDAI, patients with arthrosis, fibromyalgia, herniated disc, and depression showed higher disease activity at baseline. Of these patients, those with fibromyalgia and depression had a significantly lower clinical response (GCR) at 12 months (P < 0.05) (Table 5).

**Table 5:** Disease activity, functionality, and quality of life according to comorbidities: subgroup analysis

Comorbidity		CDAI				BASDAI				HAQ			EQ-5D		
		Baseline Mean (SD)	12 months Mean (SD)	P value	GCR (%)	Baseline Mean (SD)	12 months Mean (SD)	P value	GCR (%)	Baseline Mean (SD)	12 months Mean (SD)	P value	Baseline Mean (SD)	12 months Mean (SD)	P value
**Anxiety**	no	23.20 (17.15)	13.59 (12.34)	< 0.001	48.0	5.25 (2.52)	3.28 (2.14)	< 0.001	63.3	1.23 (0.70)	0.84 (0.59)	< 0.001	0.65 (0.18)	0.75 (0.16)	< 0.001
**n = 9**	yes	14.64 (8.48)	16.75 (15.10)	0.729	44.4	5.36 (1.97)	3.36 (1.97)	0.015	88.9	0.97 (0.61)	0.96 (0.65)	0.947	0.68 (0.11)	0.70 (0.15)	0.722
**Arthrosis**	no	22.34 (16.69)	13.61 (12.56)	< 0.001	48.5	5.24 (2.53)	3.28 (2.14)	< 0.001	63.6	1.20 (0.70)	0.83 (0.60)	< 0.001	0.65 (0.18)	0.75 (0.16)	< 0.001
**n = 7**	yes	36.57 (19.55)	17.30 (8.77)	0.069	28.6	5.63 (1.15)	3.17 (1.79)	0.043	85.7	1.86 (0.31)	1.26 (0.34)	0.019	0.52 (0.14)	0.72 (0.14)	0.021
**Depression**	no	21.37 (16.46)	12.38 (11.29)	< 0.001	**53.0** *	5.02 (2.44)	3.03 (2.05)	< 0.001	**69.0** *	1.14 (0.69)	0.78 (0.60)	< 0.001	0.67 (0.17)	0.77 (0.15)	< 0.001
**n = 37**	yes	29.36 (17.76)	19.86 (15.48)	< 0.001	**24.3** *	6.34 (2.48)	4.42 (2.10)	< 0.001	**43.2** *	1.58 (0.64)	1.17 (0.47)	< 0.001	0.53 (0.17)	0.66 (0.16)	< 0.001
**Diabetes**	no	22.39 (16.58)	13.08 (11.73)	< 0.001	48.9	5.24 (2.41)	3.28 (2.15)	< 0.001	64.2	1.20 (0.68)	0.83 (0.58)	< 0.001	0.65 (0.18)	0.75 (0.16)	< 0.001
**n = 29**	yes	25.48 (19.10)	17.67 (15.82)	0.03	41.4	5.36 (2.98)	3.28 (1.94)	< 0.001	65.5	1.36 (0.80)	0.98 (0.68)	0.008	0.62 (0.21)	0.77 (0.15)	< 0.001
**Dyslipidemia**	no	21.86 (16.29)	13.26 (12.11)	< 0.001	48.1	5.24 (2.43)	3.23 (2.17)	< 0.001	63.3	1.19 (0.69)	0.81 (0.59)	< 0.001	0.66 (0.18)	0.76 (0.16)	< 0.001
**n = 47**	yes	26.08 (18.79)	15.32 (13.52)	< 0.001	46.8	5.32 (2.71)	3.46 (1.98)	< 0.001	68.1	1.31 (0.74)	0.99 (0.59)	0.002	0.60 (0.18)	0.73 (0.15)	< 0.001
**Fibromyalgia**	no	22.38 (16.85)	13.34 (12.52)	< 0.001	**50.0** *	5.22 (2.49)	3.21 (2.12)	< 0.001	65.3	1.20 (0.69)	0.83 (0.60)	< 0.001	0.65 (0.18)	0.75 (0.16)	< 0.001
**n = 9**	yes	32.52 (16.99)	22.26 (6.53)	0.136	**0.0** *	6.06 (2.63)	4.88 (1.44)	0.211	44.4	1.74 (0.69)	1.27 (0.22)	0.058	0.55 (0.18)	0.72 (0.13)	0.039
**Gastritis**	no	22.44 (17.02)	13.40 (12.14)	< 0.001	49.0	5.18 (2.53)	3.19 (2.11)	< 0.001	**66.3** *	1.18 (0.71)	0.82 (0.59)	< 0.001	0.66 (0.18)	0.76 (0.16)	< 0.001
**n = 15**	yes	27.67 (15.61)	17.94 (15.73)	0.028	33.3	6.24 (1.76)	4.38 (2.05)	0.005	**40.0** *	1.66 (0.44)	1.22 (0.49)	0.003	0.53 (0.12)	0.65 (0.17)	0.041
**Herniated disc**	no	22.62 (16.93)	13.64 (12.50)	< 0.001	48.2	5.21 (2.51)	3.22 (2.10)	< 0.001	64.8	1.21 (0.70)	0.84 (0.60)	< 0.001	0.65 (0.18)	0.76 (0.15)	< 0.001
**n = 6**	yes	29.68 (17.34)	16.69 (11.06)	0.099	33.3	6.75 (1.45)	5.00 (2.24)	0.022	50.0	1.56 (0.56)	1.17 (0.45)	0.056	0.55 (0.11)	0.57 (0.24)	0.766
**Hypertension**	no	22.28 (16.62)	13.47 (12.29)	< 0.001	48.2	5.26 (2.50)	3.35 (2.21)	< 0.001	62.9	1.19 (0.69)	0.84 (0.59)	< 0.001	0.66 (0.18)	0.74 (0.16)	< 0.001
**n = 62**	yes	24.09 (17.74)	14.34 (12.88)	< 0.001	46.8	5.26 (2.51)	3.12 (1.91)	< 0.001	67.7	1.29 (0.73)	0.88 (0.60)	< 0.001	0.63 (0.19)	0.77 (0.14)	< 0.001
**Hypothyroidism**	no	22.54 (16.84)	13.39 (12.18)	< 0.001	49.5	5.21 (2.51)	3.26 (2.51)	< 0.001	64.1	1.21 (0.71)	0.84 (0.60)	< 0.001	0.66 (0.18)	0.75 (0.16)	< 0.001
**n = 13**	yes	27.03 (18.65)	18.82 (15.61)	0.063	23.1	6.01 (2.12)	3.61 (1.87)	< 0.001	69.2	1.30 (0.61)	0.99 (0.59)	0.025	0.52 (0.17)	0.74 (0.13)	< 0.001
**Obesity**	no	22.88 (16.90)	13.44 (12.06)	< 0.001	48.5	5.23 (2.48)	3.24 (2.13)	< 0.001	**66.2** *	1.21 (0.70)	0.84 (0.59)	< 0.001	0.65 (0.18)	0.75 (0.16)	< 0.001
**n = 7**	yes	21.41 (19.38)	21.99 (20.25)	0.903	28.6	6.05 (3.00)	4.28 (1.59)	0.096	**14.3** *	1.41 (0.83)	1.19 (0.69)	0.204	0.54 (0.27)	0.74 (0.13)	0.057

CDAI = Clinical Disease Activity Index; BASDAI = Bath Ankylosing Spondylitis Disease Activity Index; HAQ = Health Assessment Questionnaire; EQ-5D = European Quality of Life Five Dimensions; GCR = good clinical response (CDAI ≤ 10 and BASDAI < 4).

* P < 0.05.

According to the BASDAI, patients with herniated disc, depression, gastritis, fibromyalgia, obesity, and hypothyroidism had higher disease activity at baseline. Of these patients, those with depression, gastritis, and obesity had a significantly lower clinical response (GCR) at 12 months (P < 0.05) (Table 5). Furthermore, patients with arthrosis, depression, fibromyalgia, gastritis, and herniated disc had poor functionality and lower quality of life at baseline.

### Predictors of clinical response

Predictors of a worse CDAI response were female sex, comorbidities, etanercept use, and poor functionality. Predictors of a worse BASDAI response were etanercept use and poor functionality, whereas a higher quality of life was associated with a better BASDAI response. Predictors of poor functionality according to the HAQ were female sex, lower education level, and etanercept use, whereas the higher quality of life and marriage were associated with better functionality. In addition, poor functionality was a predictor of lower quality of life according to the EQ-5D (Table 6).

**Table 6:** Predictors of effectiveness, functionality, and quality of life at 12 months for bDMARD-naïve patients

CDAI response				
Predictor		β coefficient	CI 95%	P value
**HAQ**		5.91	3.24; 8.58	< 0.001
**Sex (female)**		5.39	1.55; 9.24	0.006
**Comorbidity (No)**		5.00	0.62; 9.37	0.026
**TNFi (etanercept)**		4.32	0.50; 8.15	0.027
**BASDAI response**				
**Predictor**		**β coefficient**	**CI 95%**	**P value**
**HAQ**		0.94	0.37; 1.50	0.001
**EQ-5D**		−3.33	−5.65; −1.02	0.005
**TNFi (etanercept)**		0.74	1.74; 5.87	0.014
**HAQ response**				
**Predictor**		**β coefficient**	**CI 95%**	**P value**
**EQ-5D**		−1.43	−1.88; −0.98	< 0.001
**Sex (female)**		0.26	0.9; 0.43	0.002
**Marital status (married)**		−0.22	−0.42; −0.02	0.032
**Education**				
	High school	0.23	0.04; 0.43	0.021
	Elementary	0.39	0.17; 0.61	0.001
**TNFi (etanercept)**		0.38	0.21; 0.56	< 0.001
**EQ-5D response**				
**Predictor**		β**coefficient**	**CI 95%**	**P value**
**HAQ**		−0.09	−0.11; −0.05	< 0.001

bDMARD = biologic disease-modifying antirheumatic drug; CDAI = Clinical Disease Activity Index; CI = confidence interval; HAQ = Health Assessment Questionnaire; TNFi = tumor necrosis factor inhibitor; BASDAI = Bath Ankylosing Spondylitis Disease Activity Index; EQ-5D = European Quality of Life Five Dimensions.

### Safety

The main adverse reactions reported by the patients were alopecia, headache, injection site reaction, sinusitis, flu, dyslipidemia, and infections. No cases of tuberculosis and herpes zoster were reported (Table 7).

**Table 7:** Main adverse reactions reported at 12 months by psoriatic arthritis patients who received biologic therapy

Adverse reaction	Adalimumab (91)		Etanercept (52)		Total (143)	
	n	%	n	%	n	%
Alopecia	9	9.9%	6	11.5%	15	9.7%
Headache	6	6.6%	4	7.7%	10	6.5%
Injection site reactions	5	5.5%	4	7.7%	9	5.8%
Sinusitis	4	4.4%	2	3.8%	6	3.9%
Flu	4	4.4%	1	1.9%	5	3.2%
Dyslipidemia	3	3.3%	2	3.8%	5	3.2%
Swelling	3	3.3%	1	1.9%	4	2.6%
Urinary infection	3	3.3%	1	1.9%	4	2.6%
Fungal infection	2	2.2%	2	3.8%	4	2.6%
Nausea	2	2.2%	2	3.8%	4	2.6%
Asthenia	2	2.2%	2	3.8%	4	2.6%
Brittle nails	2	2.2%	1	1.9%	3	1.9%
Dizziness	1	1.1%	1	1.9%	2	1.3%
Rhinitis	1	1.1%	1	1.9%	2	1.3%
Hypertension	2	2.2%	0	0.0%	2	1.3%
Urticaria	2	2.2%	0	0.0%	2	1.3%
Pruritus	0	0.0%	1	1.9%	1	0.6%
Diarrhea	0	0.0%	1	1.9%	1	0.6%
Weight gain	1	1.1%	0	0.0%	1	0.6%
Fever	1	1.1%	0	0.0%	1	0.6%
Others	15	16.5%	9	17.3%	24	15.5%

## DISCUSSION

This comparative study was conducted to evaluate the outcomes of PsA patients treated with adalimumab or etanercept in a real-world setting in Brazil. Loss to follow-up was 14.7% at 6 months and 35.7% at 12 months of follow-up, similar to the rates of medication non-persistence in Brazil.21 Lack of effectiveness and adverse reactions were the main causes of the loss to followup, as described in other studies.^22–24^ Although adverse reactions contributed to discontinued follow-up, the use of TNFis can be considered safe with manageable adverse reactions.^25^

Most clinical measures of disease activity, functionality, and quality of life were significantly improved at 6 and 12 months. A recent network meta-analysis reported the efficacy and acceptable safety profile of bDMARDs for PsA.^26^ Overall, TNFis could improve the signs and symptoms of articular and cutaneous involvement in addition to patient functionality and quality of life.^25–27^ Oliveira Junior et al. reported a clinical improvement in the quality of life regardless of the biologic therapy (monotherapy or combination) of patients with rheumatic diseases, including PsA. Most of the participants showed a significant clinical improvement in quality of life after 6 and 12 months of follow-up.^28^

Several comparative observational studies have been conducted for PsA and reported no differences in the effectiveness of adalimumab and etanercept, except for some outcomes such as lower medication persistence with etanercept.^4,21,24,25,29^ In our study, patients treated with adalimumab showed a greater improvement in functionality at 12 months. In addition, despite no significant differences in other outcomes, better results were obtained for disease activity and quality of life when adalimumab was administered.

In Brazil, etanercept was considered a cost-effective option in comparison to adalimumab in the past owing to its lower cost and effectiveness.^30^ However, currently, adalimumab is more cost-effective than etanercept and continues to offer some benefits, making it a cost-effective drug.^4,31^

Comorbidities, functional disability, and quality of life at baseline have been reported as predictive factors of the EQ-5D response at 12 months of follow-up.^28^ In this study, poor functionality at baseline was predictive of worse CDAI response. Studies have reported that better functionality is associated with a lower level of pain and structural damage and better work productivity, contributing to a good clinical response according to the CDAI.^32,33^ Overall, some sociodemographic and clinical factors, such as the patient's sex, marker levels, and clinical characteristics at baseline, are predictive of poor disease control over time.^34^

Despite the observed reduction in disease activity, approximately 30–50% of the patients did not achieve adequate control of PsA. A previous study reported that 45% of patients with PsA discontinued biologic therapy in the first year.^4^ Similar results have also been obtained for other rheumatic diseases.^28,35–37^ Subgroup analysis showed that patients with depression, fibromyalgia, and other comorbidities had higher disease activity in addition to poor functionality and lower quality of life at baseline. Moreover, patients with these conditions had more difficulty in achieving good control of PsA. A recent study showed that comorbidities, such as depression, fibromyalgia, obesity, and hypothyroidism, negatively affected the quality of life of patients with PsA, reducing the utility score up to 0.20.^38^

Some factors that could influence the treatment response include immunogenicity and patient preferences, which could result in a reduced clinical response.^39,40^ Patients have been found to prefer oral over injectable administration and home over hospital administration.^40,41^ These factors could affect the effectiveness of TNFis. Another factor that could influence biologic therapy is the storage of these drugs. A recent study showed that more than 80% of patients do not maintain adequate home storage conditions for biopharmaceuticals. The intrinsic factors of household refrigerators have been suggested to play a role in temperature deviations.^42^ The difficulty in the application of biopharmaceuticals by patients should be further investigated.

An important challenge faced by rheumatologists in Brazil is patient access and follow-up, which may be associated with compromised care.^43^ Furthermore, the median time to medication access through the SUS for PsA treatment following a medical prescription has been reported to be longer than 2 months.^5^ Therefore, additional strategies that can help achieve good disease control should be considered, which may include: (a) improving access to rheumatologists, which reduces the time until consultation and follow-up by a rheumatologist; (b) improving access to multidisciplinary care; (c) discovery of a novel pathway or cellular subset; (d) applying stratification biomarkers to individualize therapy; (e) preclinical intervention; (f) combination therapy with conventional synthetic drugs; (g) lifestyle modification; (h) addressing chronic pain and fatigue.^44^

A strength of this study is that this is the first comparative study carried out in a Brazilian real-life setting. Multiple outcomes were evaluated for patients diagnosed with PsA, which was conducted according to performance guidelines for evaluating incorporated drugs in the SUS to validate clinical and economic outcomes in the Brazilian population. The findings of our Brazilian study could provide useful information for health technology assessment in the SUS.

This study has some limitations. Skin involvement was not evaluated because the Brazilian clinical guidelines for PsA started to consider this manifestation only after the update in 2018.^6^ The BASDAI and CDAI are not specific indices for PsA. However, the BASDAI was used by the Brazilian clinical guidelines for PsA until 2018, and the CDAI has a strong correlation with a specific feature of PsA.^6,14^ In addition, laboratory and radiological test results were not obtained as they are not required for drug treatment through the SUS. Finally, the method used to select patients was also a limitation as only individuals who visited the health center were eligible to participate in the study. Therefore, more severe cases of PsA may not have been included, and the results should be interpreted and generalized with caution.

## CONCLUSION

The study found that TNFi therapy was effective and safe. However, despite improvements in clinical measures, most patients did not achieve adequate control of the disease, mainly those with poor functionality and lower quality of life at baseline and comorbidities, such as depression and fibromyalgia.
